# Epstein–Barr virus (EBV) and polyomaviruses are detectable in oropharyngeal cancer and EBV may have prognostic impact

**DOI:** 10.1007/s00262-020-02570-3

**Published:** 2020-04-20

**Authors:** Timo Carpén, Stina Syrjänen, Lauri Jouhi, Reija Randen-Brady, Caj Haglund, Antti Mäkitie, Petri S. Mattila, Jaana Hagström

**Affiliations:** 1 Department of Otorhinolaryngology – Head and Neck Surgery, University of Helsinki and HUS Helsinki University Hospital, P.O. Box 263, 00029 HUS Helsinki, Finland; 2 Department of Pathology, University of Helsinki and HUS Helsinki University Hospital, P.O. Box 21, 00014 HUS Helsinki, Finland; 3 Department of Oral Pathology and Oral Radiology, University of Turku and Department of Pathology, Turku University Hospital, Lemminkäisenkatu 2, 20520 Turku, Finland; 4 Department of Surgery, University of Helsinki and HUS Helsinki University Hospital, P.O. Box 440, 00029 HUS Helsinki, Finland; 5 Translational Cancer Medicine Research Program, Faculty of Medicine, University of Helsinki, P.O. Box 63, 00014 Helsinki, Finland; 6 Division of Ear, Nose and Throat Diseases, Department of Clinical Sciences, Intervention and Technology, Karolinska Institutet and Karolinska Hospital, 171 76 Stockholm, Sweden; 7 Research Program in Systems Oncology, Faculty of Medicine, University of Helsinki, Helsinki, Finland; 8 Department of Oral Pathology and Oral Radiology, University of Turku, Turku, Finland

**Keywords:** Epstein–Barr virus, Human papillomavirus, Oropharyngeal cancer, Polyomavirus, Prognosis

## Abstract

**Background:**

The etiological role of human papillomavirus (HPV) in oropharyngeal squamous cell carcinoma (OPSCC) is confirmed. However, the role of other oncoviruses in OPSCC is unknown.

**Materials and methods:**

A total of 158 consecutive OPSCC patients treated with curative intent were included. DNA extracted from tumor sections was used to detect Epstein–Barr virus (EBV), HPV, and the following polyomaviruses: John Cunningham virus (JCV), Simian virus 40 (SV40), and BK virus (BKV) with PCR. In addition, p16 expression was studied by immunohistochemistry, and EBV-encoded small RNA (EBER) transcripts were localized by in situ hybridization. The effect of viral status on overall survival (OS) and disease-free survival (DFS) was analyzed.

**Results:**

A total of 94/158 samples (59.5%) were HPV-positive, 29.1% contained BKV DNA, 20.3% EBV DNA, 13.9% JCV DNA, and 0.6% SV40 DNA. EBER was expressed only in stromal lymphocytes adjacent to the tumor and correlated with HPV positivity (*p* = 0.026). p16 expression associated only with HPV. None of the three polyomaviruses had an impact on survival. Patients with EBER-positive but HPV-negative OPSCC had significantly poorer OS and DFS than those with HPV-positive OPSCC and slightly worse prognosis compared with the patients with EBER-negative and HPV-negative OPSCC.

**Conclusion:**

Polyomaviruses are detectable in OPSCC but seem to have no impact on survival, whereas HPV was the strongest viral prognostic factor. EBER expression, as a sign of latent EBV infection, may have prognostic impact among patients with HPV-negative OPSCC. EBER analysis may identify a new subgroup of OPSCCs unrelated to HPV.

## Introduction

Several viruses have been detected in head and neck squamous cell carcinoma (HNSCC) [1–7]. Their etiologic and prognostic role is of great interest in cancer prevention and new management practices including immunotherapy [8–10] and treatment de-escalation [11–13].

The incidence of oropharyngeal squamous cell carcinoma (OPSCC) is increasing and has been attributed to human papillomavirus (HPV) infection. Currently, more than half of OPSCCs are HPV-related in many western countries, including Finland [14–17]. The prognosis of HPV-related OPSCC is more favorable than those of HPV-negative tumors [2, 15, 18, 19]. In contrast to HPV, the role of other oncogenic viruses in OPSCC is poorly understood. Depending on the endemic area, Epstein–Barr virus (EBV) is highly related to the development of nasopharyngeal carcinoma (NPC) [1, 20, 21]. EBV-associated carcinomas exhibit a non-replicating latent infection, in which translation is limited to particular genes. The gene products from latent infections, such as EBV-encoded RNAs (EBERs), are oncogenic. Importantly, patients with EBV-related NPC have a more favorable prognosis than those with EBV-negative NPC [1, 22]. Furthermore, HPV has recently been found to have a prognostic impact on NPC even in non-endemic regions [1] and co-infections with EBV and HPV have also been reported [1, 4, 22]. In OPSCC, only a few studies have detected EBV [4, 23, 24]. However, sample sizes were small in these studies. Accordingly, the prognostic role of EBV has remained unclear.

Polyomaviruses have been detected in various cancers [3, 7, 23, 25, 26]. The etiological role of Merkel cell polyomavirus in Merkel cell carcinoma is well-established [25], and various polyomaviruses have also been found in HNSCC [3, 26]. Polyomaviruses may work as cofactors in malignant transformation and tumor progression [3, 27]. Polyomaviruses BK virus (BKV) and John Cunningham virus (JCV) have been detected in the lip and laryngeal carcinoma, respectively [3]. In addition, BKV and JCV have been found in OPSCC but their impact on the outcome of OPSCC is unknown. In addition, the number of patients was small in these studies [3, 23, 24]. Polyomavirus Simian virus 40 (SV40) is related to malignancies such as osteosarcoma, non-Hodgkin lymphoma, and mesothelioma [7, 27, 28]. Additionally, SV40 has been detected in HNSCC [3] but its role is unknown.

The most recent (8th) edition of TNM classification [29, 30] uses p16 immunohistochemistry (IHC) to dichotomize OPSCC into HPV-related and -unrelated subgroups. However, it is widely known that p16 overexpression is highly sensitive but only moderately specific in HPV detection, as approximately 10% to 20% of p16 positive tumors are negative for high-risk HPV DNA [11, 31, 32]. Other oncogenic viruses can also lead to p16 overexpression in the absence of HPV [33]. Silencing of the p16 gene has been, however, also observed as a sequela of infection with oncogenic viruses other than HPV [34]. Thus, it is essential to elucidate the causative role of other viruses in OPSCC and their possible impact on p16 expression, particularly when considering treatment de-escalation for patients without conventional risk factors and suspected to have a tumor with viral etiology.

We investigated the presence of HPV and other oncoviruses including EBV and polyomaviruses (JCV, BKV, and SV40) and their association with clinicopathological variables in an OPSCC patient series. The latency of EBV infection was verified with EBER RNA expression detection. In particular, we focused on simultaneous infections with HPV and other oncoviruses. Moreover, we studied the association of these viruses with p16 overexpression and their impact on patient survival.

## Materials and methods

### Patient cohort

We identified a total of 224 consecutive OPSCC patients without previous HNSCC diagnosed between February 2012 and March 2016 at the Helsinki University Hospital, Helsinki, Finland. Patients with no available tumor tissue for p16 and viral status determination (*n* = 33) were excluded. Patients with another primary cancer at the time of OPSCC diagnosis were excluded (*n* = 4). Additionally, patients treated with palliative intent were excluded (*n* = 29). A total of 158 patients were treated with curative intent and met the inclusion criteria.

Clinical data were collected from hospital registries. Clinical- and tumor-related parameters have been discussed in part previously [14, 18, 35]. Tumor stage was determined according to the 8^th^ edition of the American Joint Committee on Cancer staging [29, 30]. Patients received either definitive radiotherapy with or without cisplatin-based chemotherapy or surgery with or without radiotherapy/chemoradiotherapy as primary treatment modalities. The follow-up schedule was the same as in our previous report [18].

### DNA extraction

DNA was extracted from tumor tissue slides by salting out the cellular proteins as described previously [3, 36]. The cell nuclei were treated in lysis buffer (10 mM Tris–HCl, 400 mM NaCl and 2 mM EDTA, pH 8.2) overnight. Proteins were digested by protease K overnight at 37 °C and then treated with saturated NaCl followed by centrifugation. Ethanol was added to the precipitated protein pellet, and DNA was extracted into a microcentrifuge tube for quantification.

### HPV genotyping, p16 immunohistochemistry, and HPV status

HPV DNA genotyping from tumor tissue slides was performed by nested PCR as described previously [14]. MY09/MY11 and GP05 +/bioGP06 + were used as external and internal primers, respectively. The genotyping was performed with a Multiplex HPV Genotyping Kit^®^ (DiaMex GmbH, Germany) that detects 24 low-risk (LR) and high-risk (HR) HPV genotypes as follows: LR-HPV6, 11, 42, 43, 44, and 70; and HR-HPV16, 18, 26, 31, 33, 35, 39, 45, 51, 52, 53, 56, 58, 59, 66, 68, 73, and 82. HPV DNA was determined as positive in a tumor sample if DNA positivity for any of the HR-HPV genotypes was detected.

p16-INK4a status was determined by IHC on paraffin-embedded formalin-fixed tissue samples as described earlier [14]. Gingival tissue was used as a positive control, and a tissue slide in diluent without primary antibody was used as a negative control. p16 expression was defined as positive if > 70% of the tumor cells had positive immunostaining. A composite variable including both p16 status and HR-HPV DNA PCR status was used to determine HPV status, as suggested by Smeets et al. [32]. Tumors both HR-HPV DNA positive and p16 positive were determined as HPV positive (HPV+) and the remaining combinations were assessed as HPV negative (HPV−).

### Detection of EBV

An in-house PCR and Luminex xMAP-based methods were used for EBV DNA detection as described in the previous studies [4, 37]. Additionally, Epstein–Barr virus (EBV)-encoded small RNA (EBER) transcripts were examined by in situ hybridization (ISH) in tumor samples to ensure the latency of EBV. Due to tumor tissue unavailability, EBER ISH was available in 89.9% (142/158) of tumor samples. EBER PNA Probe/Fluorescein and PNA-ISH Detection Kit (Dako, Glostrup, Denmark) were used for detecting EBV RNA transcripts EBER1 and EBER2 from the TMA slides. The methodology has been described in more detail in a previous study [4]. The substrate was first incubated then treated with eosin and finally mounted in Aquamount (Dako). Tumor and stromal cells were evaluated and scored separately from TMA slides by two researchers (Reija Randén-Brady and Jaana Hagström). Inconclusive cases were rescored. A total of six punches of each tumor were scored; scoring results of EBER in TMA slides were defined as follows: negative (−), mild positivity (+), moderate positivity (++), and strong positivity (+++).

### Detection of polyomaviruses

Quantitative PCR (qPCR) (Roche, Light Cycler 96, (Roche Diagnostics, Roche Molecular Diagnostics, Pleasanton, CA, UA) was used for detecting DNA of the three polyomaviruses SV40, JCV, and BKV. The primers used for amplification of all three polyomavirus T antigens and the methodology of qPCR have been described in the previous studies [3, 38]. The linear standard curves for JCV and BKV were obtained with a serial dilution of plasmids ranging from 1.2 × 10^0^ to 1.2 × 10^−2^ ng/μl for JCV and 9.5* × 10^0^ to 9.5 × 10^−3^ ng/μl for BKV as described earlier [3].

### Statistical analysis

The data were analyzed with SPSS 25 (IBM SPSS Statistics 25, IBM, Somers, IL, USA). Categorical variables were cross-tabulated using Chi square test with asymptotic and exact *p* values when best suitable. Overall survival (OS) and disease-free survival (DFS) were assessed as survival endpoints. OS was defined as the time from treatment completion to death from any cause. DFS was defined as the time from treatment completion to first recurrence or death from any cause. Survival curves were drawn using the Kaplan–Meier estimate, and the log-rank test was used to analyze the statistical significance between subgroups. The independent samples t test was performed for comparison of means of dichotomized variables. A two-sided *p* value < 0.05 was considered statistically significant.

## Results

### The presence of HPV, EBV, and polyomaviruses in tumor samples

Table 1 summarizes the prevalence of five different viral DNAs in OPSCC samples. The data on HPV have been previously published in part in a smaller patient cohort [14]. Among the viruses studied, HPV was the most prevalent, and HR-HPV DNA was detected in 97 (61.4%) tumor samples. HPV16 was the most predominant genotype detected and was in 90 (92.8%) of the HR-HPV-positive tumors, followed by four (4.1%) tumors with HPV33 and three (3.1%) with HPV18 genotypes. p16 immunopositivity was detected in 117 (74.1%) tumors. Ninety-four (59.5%) tumors were both p16- and HPV DNA-positive and were considered as HPV positive (HPV +). The remaining 64 (40.5%) tumors were considered as HPV negative. EBV DNA was detected in 32 (20.3%) tumor samples. JCV DNA was present in 22 (13.9%) and BKV DNA in 46 (29.1%) tumor samples. SV40 DNA was present only in one (0.6%) tumor sample. The viral loads of JCV in all samples were low (4.54 mean copies/100 ng DNA, SD ± 1.95), varying from 2.63 to 10.47. Higher copy numbers were detected for BKV (mean copies 19.21/100 ng DNA, median 4.22/100 ng DNA). However, the copy numbers varied widely from 2.09/100 ng DNA to 258.00/100 ng DNA.

**Table 1:** Relation of p16 and HPV DNA PCR status to different viruses

	p16+	p16−	*p*	HPV+	HPV−			p*
				HPV DNA+/p16+	HPV DNA−/p16+	HPV DNA+/p16−	HPV DNA−/p16−	
HPV DNA+	94 (96.9)	3 (3.1)	**< 0.001**					
HPV DNA−	23 (37.7)	38 (62.3)						
EBV DNA+	27 (84.4)	5 (15.6)	0.136	24 (75.0)	3 (9.4)	0 (0.0)	5 (15.6)	**0.045**
EBV DNA−	90 (71.4)	36 (28.6)		70 (55.6)	20 (15.9)	3 (2.4)	33 (26.2)	
EBER+	36 (83.7)	7 (16.3)	0.080	32 (74.4)	4 (9.3)	0 (0.0)	7 (16.3)	**0.026**
EBER−	69 (69.7)	30 (30.3)		54 (54.5)	15 (15.2)	3 (3.0)	27 (27.3)	
JCV DNA+	18 (81.8)	4 (18.2)	0.370	15 (68.2)	3 (13.6)	0 (0.0)	4 (18.2)	0.371
JCV DNA−	99 (72.8)	37 (27.2)		79 (58.1)	20 (14.7)	3 (2.2)	34 (25.0)	
BKV DNA+	34 (73.9)	12 (26.1)	0.980	27 (58.7)	7 (15.2)	1 (2.2)	11 (23.9)	0.896
BKV DNA−	83 (74.1)	29 (25.9)		67 (59.8)	16 (14.3)	2 (1.8)	27 (24.1)	
SV40 DNA+	0 (0.0)	1 (100.0)	0.259	0 (0.0)	0 (0.0)	0 (0.0)	1 (100.0)	0.405
SV40 DNA−	117 (74.5)	40 (25.5)		94 (59.9)	23 (14.6)	3 (1.9)	37 (23.6)	

*p* = *p* value

*p* values < 0.05 are bolded

*Statistical significance of comparison between HPV DNA +/p16 + group and other combinations of HPV DNA and p16

### Expression of EBER in tumor samples

**Fig. 1:**
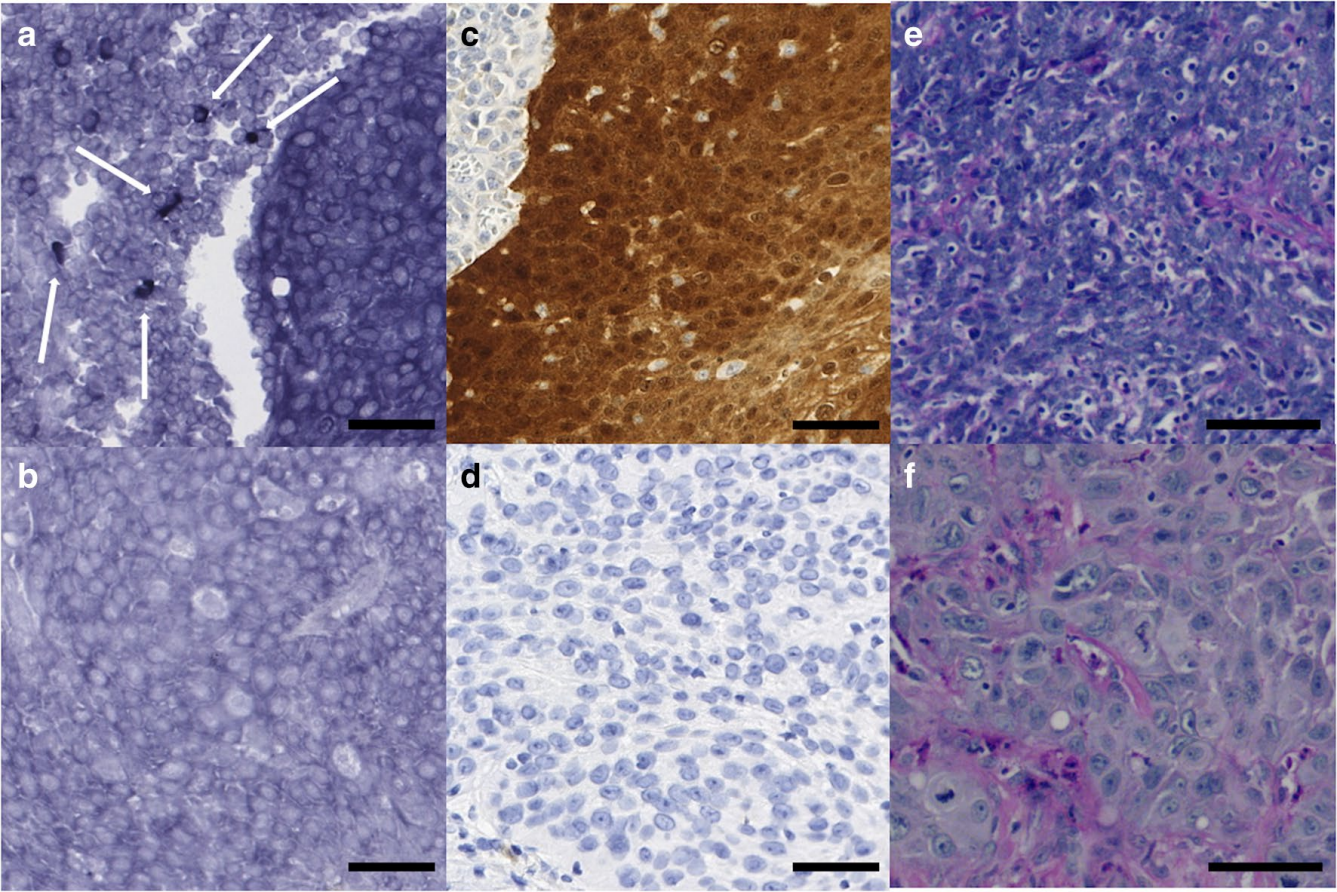
**a** Positive EBER tissue expression in the surrounding inflammatory cells of an OPSCC sample (white arrows). **b** Negative EBER tissue expression of an OPSCC sample. **c** Positive p16 tissue expression of an OPSCC sample. **d** Negative p16 tissue expression of an OPSCC sample. **e** Hematoxylin and eosin staining of a HPV-positive OPSCC sample. **f** Hematoxylin and eosin staining of a HPV-negative OPSCC sample. *Scale bar* length 50 μm. Magnifications are × 150 (**a**–**d**) and × 200 (**e**–**f**)

We detected EBER expression in the stromal inflammatory cells adjacent to the tumor invasive front in 43 (30.3%) tumor samples. EBER expression was not detectable in tumor cells (Fig. 1). EBV DNA positivity was found in tumor samples, and EBER expression detected only in the inflammatory cells correlated significantly with each other (*p* = 0.041). Among EBV DNA-positive tumor samples, half of the samples showed EBER expression (mild high); the other half was EBER negative (13/26). Among EBV-negative tumor samples, most of the samples were also EBER negative (74.1%), while 30 (25.9%) samples showed EBER expression.

### Simultaneous presence of HPV and other oncogenic viruses and their correlation with p16 overexpression in tumor samples

Coinfection with HPV was characteristic for tumors positive for other oncogenic viruses; the majority of all EBV DNA-positive (75.0%), JCV DNA-positive (68.2%), and BKV DNA-positive (58.7%) tumors were also HPV DNA- and p16-positive (Table 1). The presence of EBV DNA and EBER expression was significantly associated with HPV status.

Among the 23 p16-positive but HPV DNA-negative tumor samples, we found other viruses in nine samples. We observed EBV DNA in three tumor samples, JCV in three, and BKV in seven samples. Two of these tumors showed positivity for both EBV and BKV DNA and two for JCV and BKV DNA. Among the three HPV DNA-positive but p16-negative samples, only one sample had positivity for another oncogenic virus (BKV). Among the 38 HPV and p16-negative tumor samples, 18 tumor samples harbored DNA from other oncogenic viruses; we observed EBV DNA in five tumor samples, JCV DNA in four, BKV in 11, and SV40 in one. Two tumors showed positivity for both EBV and BKV DNA and one tumor for JCV and BKV DNA. p16 status did not have a significant association with oncogenic viruses except for HPV (Table 1).

### Only HPV status classifies OPSCC into two different entities

As reported earlier, patients with HPV-positive tumors were significantly more often male, non-smokers, and diagnosed with disease extended to local lymph nodes compared with HPV-negative tumors [14]. In addition, patients with HPV-negative tumors were significantly more often heavy alcohol users and had a higher stage (III–IV) compared with patients with HPV-positive tumors. Part of the clinicopathological results according to patients with HPV-positive and HPV-negative tumors has been presented previously in a smaller patient cohort [14]. Patients with EBV DNA-positive tumors had significantly more often stage I–II disease compared with those with EBV DNA-negative tumors. EBER-positive tumors were more frequently smaller in T-class (T1–T2) and had a high propensity to localize in tonsils, whereas EBER-negative tumors were more often advanced (T3–T4) and had a less imbalanced distribution in oropharyngeal sublocalizations. None of the polyomaviruses had an impact on any of the clinicopathological factors. The differences in clinicopathological factors between different viruses are shown in Table 2.

**Table 2:** Demographic and tumor-related factors according to HPV, EBV DNA, EBER, JCV DNA, and BKV DNA status

Variable	HPV+*	HPV−**	*p*	EBV DNA+	EBV DNA−	*p*	EBER+	EBER−	*p*	JCV DNA+	JCV DNA−	*p*	BKV DNA+	BKV DNA−	*p*
Mean age	60.8	62.1	0.400	61.4	61.3	0.969	62.5	61.0	0.360	62.6	61.2	0.499	60.5	61.7	0.445
Sex			**0.033**			0.126			0.663			0.876			0.229
Male	77 (81.9)	43 (67.2)		21 (65.6)	99 (78.6)		32 (74.4)	77 (77.8)		17 (77.3)	103 (75.7)		32(69.6)	88 (78.6)	
Female	17 (18.1)	21 (32.8)		11 (34.4)	27 (21.4)		11 (25.6)	22 (22.2)		5 (22.7)	33 (24.3)		14 (30.4)	24 (21.4)	
Smoking			**< 0.001**			0.213			0.723			0.870			0.256
Non-smoker	33 (35.1)	10 (15.6)		11 (34.4)	32 (25.4)		13 (30.2)	27 (27.3)		5 (22.7)	38 (27.9)		15 (32.6)	28 (25.0)	
Ex-smoker	39 (41.5)	10 (15.6)		12 (37.5)	37 (29.4)		14 (32.6)	28 (28.3)		7 (31.8)	42 (30.9)		10 (21.7)	39 (34.8)	
Current smoker	22 (23.4)	44 (68.8)		9 (28.1)	57 (45.2)		16 (37.2)	44 (44.4)		10 (45.5)	56 (41.2)		21 (45.7)	45 (40.2)	
Heavy alcohol use			**0.001**			0.070			0.419			0.360			0.878
None	52 (72.2)	24 (40.0)		16 (69.6)	60 (55.0)		17 (56.7)	51 (57.3)		14 (70.0)	62 (55.4)		24 (60.0)	52 (56.5)	
Former	6 (8.3)	13 (21.7)		0 (0)	19 (17.4)		6 (20.0)	10 (11.2)		3 (15.0)	16 (14.3)		6 (15.0)	13 (14.1)	
Current	14 (19.4)	23 (38.3)		7 (30.4)	30 (27.5)		7 (23.3)	28 (31.5)		3 (15.0)	34 (30.4)		10 (25.0)	27 (29.3)	
T class			0.925			0.192			**0.049**			0.751			0.268
T1–T2	61 (64.9)	42 (65.6)		24 (75.0)	79 (62.7)		33 (76.7)	59 (59.6)		15 (68.2)	88 (64.7)		33 (71.7)	70 (62.5)	
T2–T3	33 (35.1)	22 (34.4)		8 (25.0)	47 (37.3)		10 (23.3)	40 (40.4)		7 (31.8)	48 (35.3)		13 (28.3)	42 (37.5)	
N class			**0.002**			0.421			0.087			0.769			0.183
N0	9 (9.6)	18 (28.1)		7 (21.9)	20 (15.9)		4 (9.3)	21 (21.2)		3 (13.6)	24 (17.6)		5 (10.9)	22 (19.6)	
N+	85 (90.4)	46 (71.9)		25 (78.1)	106 (84.1)		39 (90.7)	78 (78.8)		19 (86.4)	112 (82.4)		41 (89.1)	90 (80.4)	
Clinical stage			**< 0.001**			**0.024**			0.069			0.127			0.420
I–II	75 (79.8)	32 (50.0)		27 (84.4)	80 (63.5)		34 (79.1)	63 (63.6)		18 (81.8)	89 (65.4)		29 (63.0)	78 (69.6)	
III–IV	19 (20.2)	32 (50.0)		5 (15.6)	46 (36.5)		9 (20.9)	36 (36.4)		4 (18.2)	47 (34.6)		17 (37.0)	34 (30.4)	
Tumor site			**< 0.001**			0.085			**0.002**			0.770			0.207
Tonsil	69 (73.4)	26 (40.6)		22 (68.8)	73 (57.9)		36 (83.7)	50 (50.5)		14 (63.6)	81 (59.6)		28 (60.9)	67 (59.8)	
Base of tongue	24 (25.5)	18 (28.1)		9 (28.1)	33 (26.2)		4 (9.3)	33 (33.3)		6 (27.3)	36 (26.5)		13 (28.3)	29 (25.9)	
Soft palate	1 (1.1)	15 (23.4)		1 (3.1)	15 (11.9)		2 (4.7)	12 (12.1)		1 (4.5)	15 (11.0)		2 (4.3)	14 (12.5)	
Posterior wall	0 (0)	5 (7.8)		0 (0)	5 (4.0)		1 (2.3)	4 (4.0)		1 (4.5)	4 (2.9)		3 (6.5)	2 (1.8)	
Grade			**< 0.001**			0.754			0.346			0.491			0.854
I	0 (0)	4 (6.3)		0 (0)	4 (3.2)		0 (0)	4 (4.0)		0 (0)	4 (2.9)		1 (2.2)	3 (2.7)	
II	7 (7.4)	21 (32.8)		5 (15.6)	23 (18.3)		6 (14.0)	19 (19.2)		2 (9.1)	26 (19.1)		7 (15.2)	21 (18.8)	
III	87 (92.6)	39 (60.9)		27 (84.4)	99(78.6)		37 (86.0)	76(76.8)		20 (90.9)	106 (77.9)		38 (82.6)	88 (78.6)	
Treatment			0.273			0.885			0.265			0.481			0.420
RT/CRT	61 (64.9)	36 (56.3)		20 (62.5)	77 (61.1)		24 (55.8)	65 (65.7)		15 (68.2)	82 (60.3)		26 (56.5)	71 (63.4)	
Sx ± RT/CRT	33 (35.1)	28 (43.8)		12 (37.5)	49 (38.9)		19 (44.2)	34 (34.3)		7 (31.8)	54 (39.7)		20 (43.5)	41 (36.6)	

*SX* surgery, *RT* radiotherapy, *CRT* chemoradiotherapy. N+ = extend to cervical lymph nodes

*p* = *p* value. Chi square test was used for cross tabulation and Fisher’s Exact Test when needed. *p* values < 0.05 are bolded. Percentagesmay not addup to 100 because of rounding

*Tumors being both p16 positive and HPV DNA positive were considered as HPV+

**The rest of the HPV-DNA and p16 combinations were determined as HPV−

### The impact of different viruses on prognosis

**Fig. 2:**
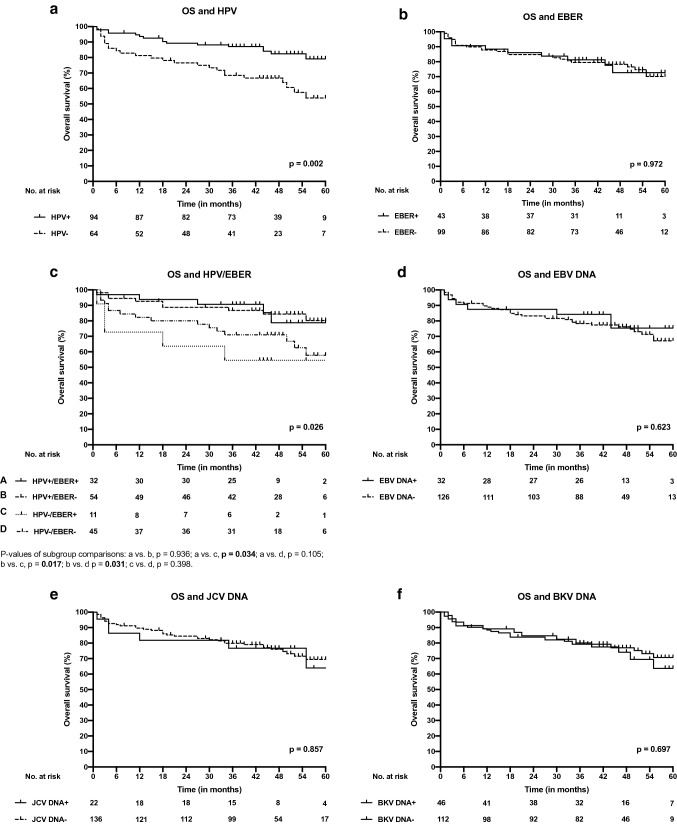
Overall survival (OS) curves according to different viruses in OPSCC. **a** OS of patients with HPV-positive and HPV-negative OPSCC. **b** OS of patients with EBER-positive and EBER-negative OPSCC. **c** OS of patients with different combinations of EBER and HPV in OPSCC. **d** OS of patients with EBV DNA-positive and EBV DNA-negative OPSCC. **e** OS of patients with JCV DNA-positive and JCV DNA-negative OPSCC and **f** OS of patients with BKV-positive and BKV-negative OPSCC

**Fig. 3:**
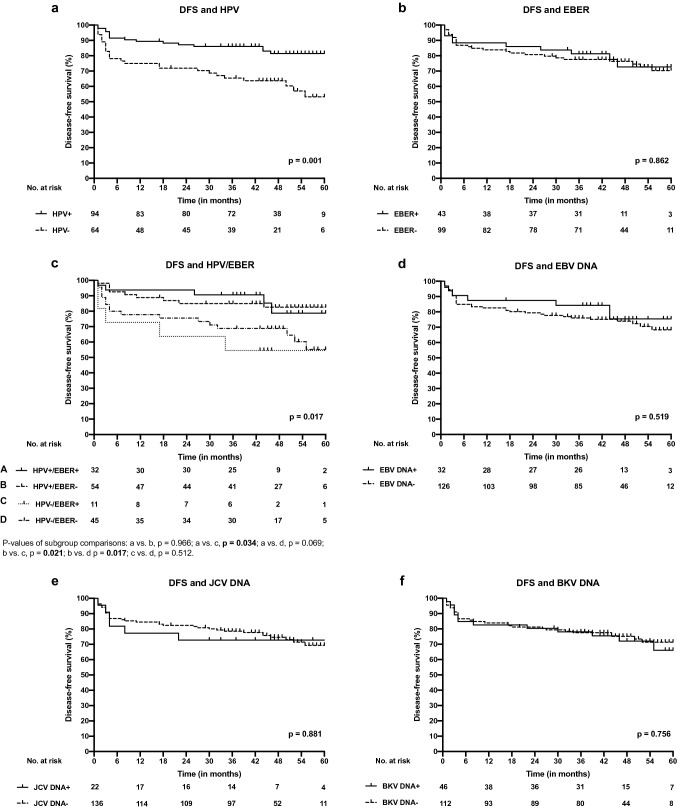
Disease-free survival (DFS) curves according to different viruses in OPSCC. **a** DFS of patients with HPV-positive and HPV-negative OPSCC. **b** DFS of patients with EBER-positive and EBER-negative OPSCC. **c** DFS of patients with different combinations of EBER and HPV in OPSCC. **d** DFS of patients with EBV DNA-positive and EBV DNA-negative OPSCC. **e** DFS of patients with JCV DNA-positive and JCV DNA-negative OPSCC and **f** DFS of patients with BKV-positive and BKV-negative OPSCC

The median follow-up time of the patients was 46 months (range 0–66 months). Patients with HPV-positive tumors had a significantly more favorable OS (*p* = 0.002) and DFS (*p* = 0.001) compared with HPV-negative tumors. EBV, EBER, JCV, or SV40 did not have a significant impact on OS (Fig. 2) or DFS (Fig. 3) when compared independently. As HPV positivity was significantly related to EBER positivity, we compared the differences in patient survival between patients with different combinations of HPV and EBER tumors. OS and DFS did not differ significantly between patients carrying HPV and EBER-negative OPSCC and HPV-positive OPSCC regardless of EBER expression. Patients with HPV-negative but EBER-positive OPSCC had significantly poorer OS and DFS when compared with patients carrying HPV-positive OPSCC regardless of EBER expression (Figs. 2 and 3).

## Discussion

This study provides new evidence on the presence of EBV and the polyomaviruses JCV, BKV, and SV40 in OPSCC. Our results also support the paradigm of HPV as the predominant virus in OPSCC. Although several viruses have been detected in HNSCC, the most important viruses are HPV in OPSCC and EBV in NPC [1–3, 5, 14, 26]. The etiologic role of HPV and EBV in the pathogenesis and prognosis of OPSCC and NPC, respectively, is well-established [1, 2, 19, 21]. However, there are only limited data on other oncoviruses except HPV in the etiopathogenesis of OPSCC. To our knowledge, this is the first study focusing not only on the detection but also the prognostic role of other oncogenic viruses in OPSCC.

Our analysis revealed EBER expression in approximately one-third of OPSCC samples. Instead of being present in tumor cells, EBER was expressed only in the stromal inflammatory cells closest to the tumor invasive front. Interestingly, EBER expression correlated significantly with tumor HPV positivity. The positive EBER expression in different HNSCCs (including a few OPSCC samples) has been presented recently [4]. Although the presence of HPV/EBV coinfection in OPSCC has been reported previously [39, 40], we are the first to show a significant correlation between EBER positivity and HPV positivity in OPSCC in a relatively large patient cohort. Recently, it has been reported that EBERs, which are the sign of latent EBV infection, are secreted from EBV-infected cells and are recognized by toll-like receptor 3. This recognition leads to the induction of type I IFNs and inflammatory cytokines and subsequent immune activation [41, 42]. Thus, lymphocytes carrying latent EBV infection might aid immune activation as the EBER positivity in inflammatory cells can be interpreted as cleared EBV infection of tumor cells [4]. It has been suggested that the presence of EBV may increase the invasiveness of HPV-positive OPSCC tumors [39]. On the other hand, the presence of EBV is more related to epithelial dysplasia and precancerous tissue than to a higher stage of a malignant disease [43]. In our study, EBER positivity was related to lower T class and EBV DNA positivity to the lower stage. This may be due to the fact that the majority of EBER-positive and EBV DNA-positive tumors were also HPV positive; HPV-positive tumors are generally present with lower T class and are classified at a lower stage (I-II) according to the newest TNM classification [18, 29]. Neither EBER nor EBV DNA positivity had an impact on prognosis when compared with patients with EBER-negative and EBV-negative tumors, respectively. However, it appeared that among the HPV-negative subgroup, those with EBER-positive tumors had significantly poorer outcomes than patients with HPV-positive tumors. To our knowledge, this has not been reported previously for OPSCC. In NPC, EBV has a clear prognostic role and EBV-specific immunotherapy has recently showed promising results [1, 44, 45]. According to our results, EBV may have also prognostic impact on OPSCC. Therefore, its prognostic significance in OPSCC should be studied more profoundly in the future. Regarding EBV and HPV synergy, it has been speculated that HPV may inhibit EBV lytic replication, facilitating the establishment of EBV latency [46]. EBER positivity is associated with poorer survival in HNSCC [4] even though its favorable impact on the prognosis of NPC is well-established [1]. Our analysis suggests that EBV may act as a cofactor in HPV-associated OPSCC, as previously suggested [39, 46]. In addition, EBV-specific immunotherapy has showed promising results in NPC [44, 45]. According to our results, EBV may have a prognostic impact on OPSCC and should be taken into account in future study designs and treatment approaches.

Different polyomaviruses, including JCV, BKV, and SV40 have been proposed to act as cofactors in oncogenic transformation and tumor progression in different cancers [3, 7]. Here we detected BKV DNA in 46 tumors (29%). This amount is higher than previously reported in HNSCC, particularly in OPSCC [3, 24]. JCV DNA was present in 13.9% of our OPSCC samples. JCV has also been detected in pharyngeal carcinoma (site not specified) with higher copy numbers than found in corresponding normal tissue, although the numbers analyzed are still limited [3, 47]. The presence of BKV and JCV DNA in OPSCC was expected as these viruses are known to infect B lymphocytes, which are also abundantly present in the oropharynx [38, 48]. We found that coinfections as part of HPV-positive tumors were additionally positive for BKV DNA and JCV DNA. However, in our series positivity for neither BKV nor JCV yielded any statistically significant differences in clinicopathological characteristics when compared with BKV-negative and JCV-negative patients, respectively. Only one tumor sample was positive for SV40DNA. SV40 DNA has been detected previously in different HNSCCs, such as lip and larynx cancer, but not in OPSCC [3]. Our results thus indicate that the role of polyomaviruses in OPSCC seems to be insignificant.

The relationship between p16 immunopositivity and HPV DNA presence in OPSCC is well-established [49]. The most recent TNM classifications stratify OPSCC into two disease entities according to p16 status [29, 30], despite its limitations as a standalone surrogate marker for HPV involvement in OPSCC [32]. In addition to HPV, other oncoviruses that interact with the retinoblastoma (pRb) pathway have been suggested to cause p16 overexpression in OPSCC [33]. However, there may be other mechanisms, such as point mutations and gene deletions that lead to inactivation of pRb and overexpression of the p16 protein [11]. As the other HPV-independent factors that potentially lead to p16 overexpression are poorly understood, our objective was to evaluate the association between p16 immunopositivity and the presence of the viruses studied here. We found EBV, JCV, and BKV DNA in a subgroup of p16-positive but HPV-negative tumors. Thus, p16 may be overexpressed due to other viruses independently of HPV. Previously, it has been shown that the T-antigen of JCV and BKV interacts with pRb and may lead to inactivation of pRb [50]. EBV infection has been found to decrease expression of pRb [51, 52]. These findings may partly explain the elevated p16 expression in JCV, BKV, and EBV DNA-positive tumors. However, the majority of the tumors that were solely EBV, JCV, or BKV positive were p16 negative.

As expected, we also confirmed that over half (59.5%) of the OPSCCs associated with HPV, consistent with previous observations in Finland [15]. Our analysis also confirmed the close association between p16 immunopositivity and HPV presence in OPSCC tumors. Consistent with the previous reports, patients with HPV-positive OPSCC had significant differences in patient- and tumor-related factors compared with HPV-negative OPSCC patients [2, 14, 53, 54]. Patients with HPV-positive OPSCC had significantly more favorable OS and DFS compared with their HPV-negative counterparts, as established earlier [2, 14, 15, 19].

This study has some limitations. Alcohol use was not known for every patient and EBER ISH was not available for all patients due to the absence of tumor samples or tumor tissue of insufficient size. The strengths of the present study are the relatively large patient cohort with long follow-up time. In addition, p16 status and viral DNA status could be determined for each tumor sample, thus allowing comprehensive analyses.

## Conclusions

We showed that polyomaviruses are detectable in OPSCC but seem to have no association with clinicopathological features or prognosis. EBER RNA expression was not found in tumor cells but rather in the stromal lymphocytes adjacent to the invasive front. EBER expression was associated with HPV positivity. However, EBER expression in HPV-negative OPSCC had tendency to associate with poorer survival when compared with patients with HPV-negative and EBER-negative OPSCC, which to our knowledge is a novel observation. HPV was the only virus that had significant impact on prognosis and correlated significantly with p16 status. HPV presence in our series divided OPSCC into two disease entities according to clinicopathological factors. However, EBER analysis might identify a new subgroup of patients with OPSCC that is not related to HPV.

## Abbreviations

BKV: BK virus
DFS: Disease-free survival
EBER: Epstein–Barr virus (EBV)-encoded small RNA
EBV: Epstein–Barr virus
HNSCC: Head and neck squamous cell carcinoma
IHC: Immunohistochemistry
ISH: In situ hybridization
JCV: John Cunningham virus
NPC: Nasopharyngeal carcinoma
OPSCC: Oropharyngeal squamous cell carcinoma
OS: Overall survival
qPCR: Quantitative polymerase chain reaction
SV40: Simian virus 40
